# *N*,*N*-Bis(2-hy­droxy­eth­yl)-4-methyl­benzene­sulfonamide

**DOI:** 10.1107/S1600536812041682

**Published:** 2012-10-20

**Authors:** Nafeesa Mushtaq, Islam Ullah Khan, Muhammad Yar, Sadia Afzal, Jim Simpson

**Affiliations:** a Materials Chemistry Laboratory, Department of Chemistry, GC University, Lahore 54000, Pakistan; b Department of Chemistry, University of Otago, PO Box 56, Dunedin 9054, New Zealand

## Abstract

In the title compound C_11_H_17_NO_4_S, an intra­molecular O—H⋯O hydrogen bond forms an *S*(8) ring and determines the conformation of the bis­(2-hy­droxy­eth­yl) segment of the mol­ecule, holding the two CH_2_CH_2_OH groups close to coplanar (r.m.s. deviation = 0.185 Å). In the crystal, O—H⋯O hydrogen bonds link the mol­ecules into zigzag chains along the *b* axis. Weaker additional C—H⋯O and C—H⋯π contacts generate a three dimensional network, with mol­ecules stacked along the *b*-axis direction.

## Related literature

For pharmaceutical background to sulfonamides, see: Casini *et al.* (2002 ▶); Chambers & Jawetz (1998 ▶). For an alternative synthesis, see: Hori *et al.* (2011 ▶). For a related structure, see: Yoon *et al.* (2001 ▶). For standard bond lengths, see: Allen *et al.* (1987 ▶) and for hydrogen-bond motifs, see: Bernstein *et al.* (1995 ▶). 
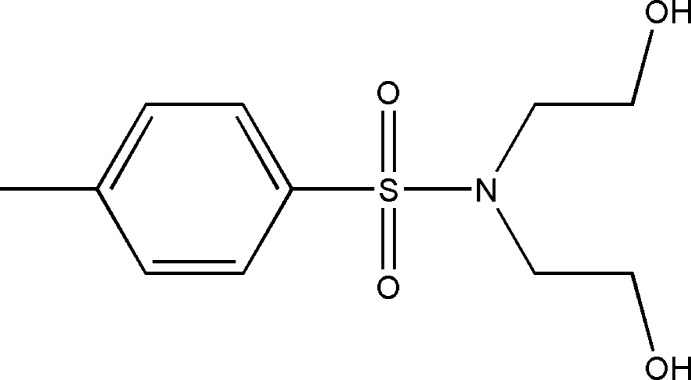


## Experimental

### 

#### Crystal data

C_11_H_17_NO_4_S
*M* *_r_* = 259.32
Orthorhombic, 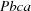

*a* = 17.9308 (5) Å
*b* = 7.1881 (2) Å
*c* = 19.8333 (6) Å
*V* = 2556.28 (13) Å^3^
*Z* = 8
Mo *K*α radiation
μ = 0.26 mm^−1^
*T* = 296 K
0.16 × 0.12 × 0.10 mm

#### Data collection

Bruker Kappa APEXII CCD diffractometer
3124 measured reflections
3124 independent reflections
2343 reflections with *I* > 2σ(*I*)

#### Refinement

*R*[*F* ^2^ > 2σ(*F* ^2^)] = 0.050
*wR*(*F* ^2^) = 0.161
*S* = 0.99
3124 reflections
161 parameters
H atoms treated by a mixture of independent and constrained refinement
Δρ_max_ = 0.45 e Å^−3^
Δρ_min_ = −0.39 e Å^−3^

### 

Data collection: *APEX2* (Bruker, 2007 ▶); cell refinement: *APEX2* and *SAINT* (Bruker, 2007 ▶); data reduction: *SAINT*; program(s) used to solve structure: *SHELXS97* (Sheldrick, 2008 ▶); program(s) used to refine structure: *SHELXL97* (Sheldrick, 2008 ▶); molecular graphics: *ORTEP-3* (Farrugia, 1997 ▶) and *Mercury* (Macrae *et al.*, 2008 ▶); software used to prepare material for publication: *SHELXL97*, *ORTEP-3* (Farrugia, 1997 ▶), *enCIFer* (Allen *et al.*, 2004 ▶), *PLATON* (Spek, 2009 ▶) and *publCIF* (Westrip 2010 ▶).

## Supplementary Material

Crystal structure: contains datablock(s) I, New_Global_Publ_Block. DOI: 10.1107/S1600536812041682/nk2181sup1.cif

Structure factors: contains datablock(s) I. DOI: 10.1107/S1600536812041682/nk2181Isup2.hkl

Additional supplementary materials:  crystallographic information; 3D view; checkCIF report

## Figures and Tables

**Table 1:** Hydrogen-bond geometry (Å, °) *Cg*1 is the centroid of the C1–C6 benzene ring.

*D*—H⋯*A*	*D*—H	H⋯*A*	*D*⋯*A*	*D*—H⋯*A*
O4—H4*O*⋯O3	0.76 (7)	1.89 (7)	2.634 (3)	166 (7)
O3—H3*O*⋯O4^i^	0.75 (4)	1.92 (4)	2.661 (3)	172 (4)
C10—H10*A*⋯O1^ii^	0.97	2.61	3.361 (3)	135
C3—H3⋯*Cg*1^iii^	0.93	2.78	3.522 (2)	138

Symmetry codes: (i) 
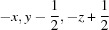
; (ii) 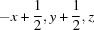; (iii) 
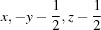
.

**Fig. 1.**
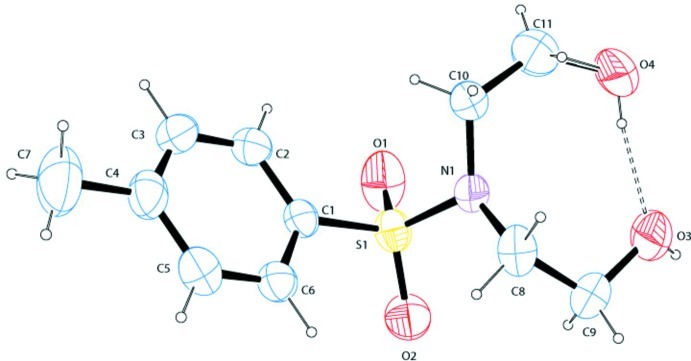
The molecular structure of (1) showing the atom numbering scheme with displacement ellipsoids drawn at the 50% probability level. The intramolecular hydrogen bond is drawn as an open dashed line.

**Fig. 2.**
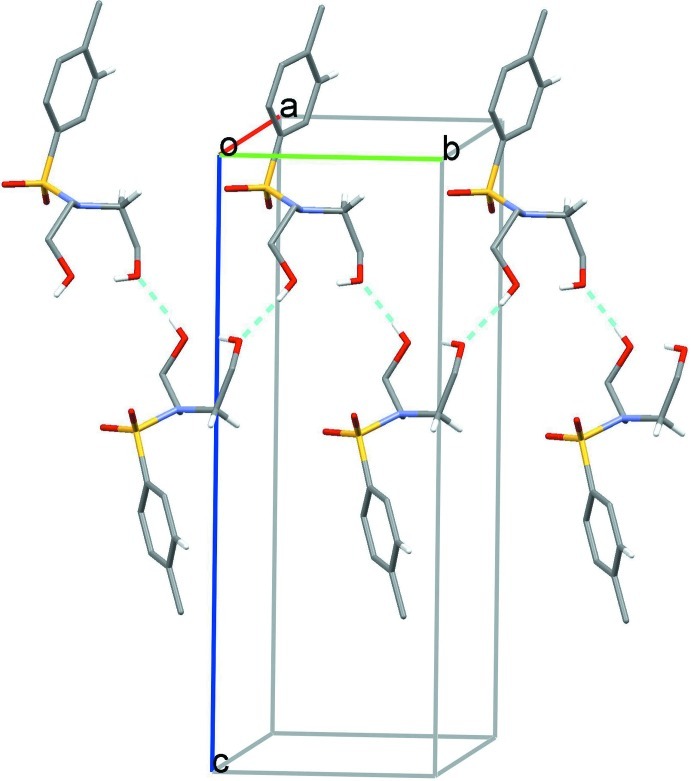
Zigzag chains of molecules formed along b by intermolecular O—H···O hydrogen bonds, shown as dashed lines.

**Fig. 3.**
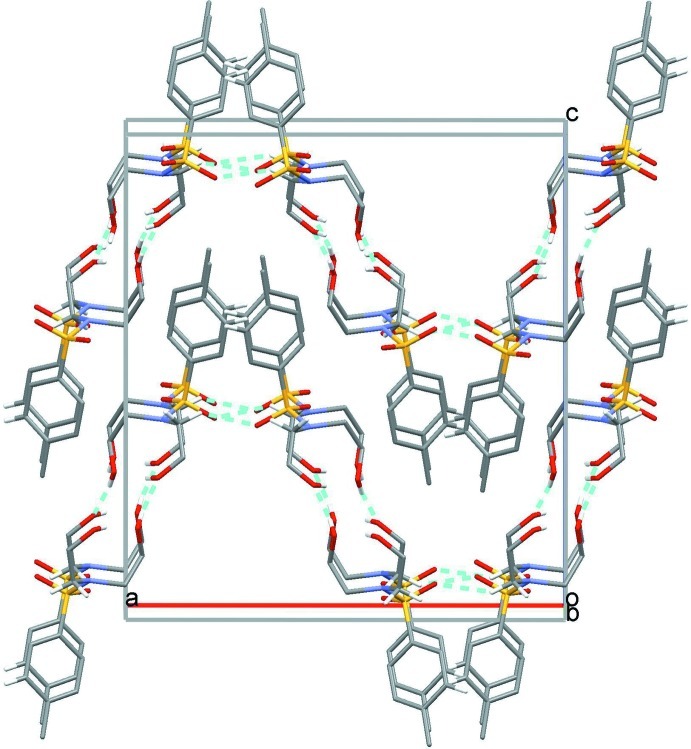
Overall packing for (1) viewed along the b axis with hydrogen bonds drawn as dashed lines.

## supplementary crystallographic information

### Comment

Sulfonamides are an important class of drugs with antibacterial, diuretic, hypoglycemic, antithyroid and antitumor action (Casini *et al.*, 2002). Certain sulfonamides are used in combination with other drugs as antimicrobial agents and this paved the way for an antibiotic revolution in medicine. Sulfonamides also act as anti-metabolites and compete for the enzyme involved in the production of folic acid (Chambers & Jawetz, 1998). Most sulfonamides behave as weak acids and binding to basic amino acids can occur. The crystal structure of a tritosylate of diethanolamine has been reported in which the three tosyl groups are separated from one another as much as possible, to minimize steric repulsion (Yoon *et al.*, 2001). The promising pharmaceutical potential of sulfonamides has prompted us to synthesize and characterize diethanolamine derived sulfonamides. Diethanolamine (DEA) itself has skin irritant characteristics; however, when DEA is transformed into new materials, the resulting compounds may become pharmaceutically useful. We therefore synthesized *N*,*N*-bis(2-hydroxyethyl)-4-methylbenzenesulfonamide (*N*-tosyl diethanolamine) (1) and report its molecular and crystal structure here.

The molecular structure of (1) is shown in Fig. 1. The tolyl ring (C1···C6) is inclined at dihedral angles of 27.29 (0.16) ° and 37.78 (0.17) ° with respect to the N1/C8/C9 and N1/C10/C11 planes respectively. Bond distances (Allen *et al.*, 1987) and angles within the molecule are normal and similar to those reported for *N*,*N*-bis(tosyloxyethyl)-*p*-toluenesulfonamide (Yoon *et al.*, 2001). The nitrogen atom of the diethanolamine substituent is approximately *sp*^3^ hybridized with the geometry around the N1 atom close to pyramidal [C8—N1—S1 = 117.49 (14)°, C10—N1—S1 = 116.76 (14)° and C8—N1—C10 = 117.98 (18)°]. The two ethanol substituents are unsymmetrically oriented around the nitrogen atom with their conformations ultimately determined by an intramolecular O4—H4···O3 hydrogen bond that forms an S(8) ring (Bernstein *et al.*, 1995).

In the crystal structure intermolecular O3—H3···O4 hydrogen bonds, Table 1, link molecules into zigzag chains along the *b* axis, Fig 2. C3—H3···π contacts, together with additional C10—H10A···O1 hydrogen bonds, further connect the chains to form a three-dimensional network structure, with molecules stacked along *b*, Fig 3.

### Experimental

Tosyl chloride (0.836 g, 4.4 mmol) was added to a flask containing diethanolamine (0.42 g, 4 mmol) dissolved in CH_2_Cl_2_. Pyridine was added as a base (0.35 g, 4.4 mmol) and the solution refluxed for 4 hrs. On completion of the reaction, the pyridine was removed *in vacuo* and the product purified by crystallization. White crystals were obtained with an overall yield of 77%; Rf 0.13 (1:1 hexane and ethyl acetate). M.P: 95–98 0 C; 1H NMR 500 MHz, CDCl_3_: 7.70 (d, 2H), 7.33 (d, 2H), 3.87 (t, –CH_2_OH, 4H), 3.23 (t, –CH_2_N–, 4H), 2.43 (s, –CH_3_, 3H). The synthesis of (1) has also been reported using triethylamine rather than pyridine (Hori *et al.*, 2011).

### Refinement

H atoms of the OH groups were located in a difference Fourier map and their coordinates refined with *U*_eq_ = 1.5*U*_eq_ (O). Other H-atoms were refined using a riding model with d(C—H) = 0.93 Å for aromatic, 0.97 Å and for CH_2_ H atoms with *U*_iso_ = 1.2*U*_eq_ (C) and 0.96 Å, *U*_iso_ = 1.5*U*_eq_ (C) for CH_3_ H atoms.

### Crystal data

**Table:**

C_11_H_17_NO_4_S	*F*(000) = 1104
*M**_r_* = 259.32	*D*_x_ = 1.348 Mg m^−^^3^
Orthorhombic, *P**b**c**a*	Mo *K*α radiation, λ = 0.71073 Å
Hall symbol: -P 2ac 2ab	Cell parameters from 4787 reflections
*a* = 17.9308 (5) Å	θ = 2.3–27.7°
*b* = 7.1881 (2) Å	µ = 0.26 mm^−^^1^
*c* = 19.8333 (6) Å	*T* = 296 K
*V* = 2556.28 (13) Å^3^	Block, colourless
*Z* = 8	0.16 × 0.12 × 0.10 mm

### Data collection

**Table:**

Bruker Kappa APEXII CCD diffractometer	2343 reflections with *I* > 2σ(*I*)
Radiation source: fine-focus sealed tube	*R*_int_ = 0.000
Graphite monochromator	θ_max_ = 28.3°, θ_min_ = 3.1°
φ and ω scans	*h* = 0→23
3124 measured reflections	*k* = 0→9
3124 independent reflections	*l* = 0→25

### Refinement

**Table:**

Refinement on *F*^2^	Primary atom site location: structure-invariant direct methods
Least-squares matrix: full	Secondary atom site location: difference Fourier map
*R*[*F*^2^ > 2σ(*F*^2^)] = 0.050	Hydrogen site location: inferred from neighbouring sites
*wR*(*F*^2^) = 0.161	H atoms treated by a mixture of independent and constrained refinement
*S* = 0.99	*w* = 1/[σ^2^(*F*_o_^2^) + (0.0948*P*)^2^ + 1.0844*P*] where *P* = (*F*_o_^2^ + 2*F*_c_^2^)/3
3124 reflections	(Δ/σ)_max_ = 0.009
161 parameters	Δρ_max_ = 0.45 e Å^−^^3^
0 restraints	Δρ_min_ = −0.39 e Å^−^^3^

### Special details

**Table:**

Geometry. All e.s.d.'s (except the e.s.d. in the dihedral angle between two l.s. planes) are estimated using the full covariance matrix. The cell e.s.d.'s are taken into account individually in the estimation of e.s.d.'s in distances, angles and torsion angles; correlations between e.s.d.'s in cell parameters are only used when they are defined by crystal symmetry. An approximate (isotropic) treatment of cell e.s.d.'s is used for estimating e.s.d.'s involving l.s. planes.
Refinement. Refinement of *F*^2^ against ALL reflections. The weighted *R*-factor *wR* and goodness of fit *S* are based on *F*^2^, conventional *R*-factors *R* are based on *F*, with *F* set to zero for negative *F*^2^. The threshold expression of *F*^2^ > σ(*F*^2^) is used only for calculating *R*-factors(gt) *etc*. and is not relevant to the choice of reflections for refinement. *R*-factors based on *F*^2^ are statistically about twice as large as those based on *F*, and *R*- factors based on ALL data will be even larger.

### Fractional atomic coordinates and isotropic or equivalent isotropic displacement parameters (Å^2^)

**Table:**

	*x*	*y*	*z*	*U*_iso_*/*U*_eq_	
O1	0.20186 (10)	0.1722 (3)	0.09437 (9)	0.0733 (6)	
O2	0.08536 (10)	0.0146 (2)	0.06009 (8)	0.0598 (4)	
S1	0.13232 (3)	0.17523 (7)	0.05993 (3)	0.04344 (19)	
C1	0.15067 (10)	0.2344 (3)	−0.02460 (10)	0.0382 (4)	
C2	0.21585 (12)	0.3277 (3)	−0.04138 (12)	0.0501 (5)	
H2	0.2511	0.3542	−0.0084	0.060*	
C3	0.22799 (14)	0.3806 (3)	−0.10709 (13)	0.0575 (6)	
H3	0.2716	0.4437	−0.1181	0.069*	
C4	0.17630 (14)	0.3417 (3)	−0.15754 (12)	0.0529 (6)	
C7	0.1893 (2)	0.4011 (5)	−0.22931 (15)	0.0846 (9)	
H7A	0.2302	0.3318	−0.2478	0.127*	
H7B	0.2008	0.5315	−0.2305	0.127*	
H7C	0.1452	0.3778	−0.2554	0.127*	
C5	0.11233 (13)	0.2452 (3)	−0.13986 (11)	0.0501 (5)	
H5	0.0777	0.2156	−0.1731	0.060*	
C6	0.09883 (11)	0.1920 (3)	−0.07401 (10)	0.0420 (4)	
H6	0.0553	0.1284	−0.0630	0.050*	
N1	0.08353 (9)	0.3445 (2)	0.09165 (9)	0.0429 (4)	
C8	0.00254 (12)	0.3381 (3)	0.08243 (11)	0.0495 (5)	
H8A	−0.0087	0.2717	0.0411	0.059*	
H8B	−0.0162	0.4640	0.0777	0.059*	
C9	−0.03700 (14)	0.2449 (4)	0.14009 (13)	0.0635 (7)	
H9A	−0.0899	0.2359	0.1302	0.076*	
H9B	−0.0177	0.1200	0.1460	0.076*	
O3	−0.02636 (12)	0.3483 (3)	0.19990 (9)	0.0680 (6)	
H3O	−0.036 (2)	0.286 (6)	0.2288 (19)	0.102*	
C10	0.11973 (15)	0.5289 (3)	0.09459 (12)	0.0600 (7)	
H10A	0.1681	0.5203	0.0729	0.072*	
H10B	0.0899	0.6163	0.0689	0.072*	
C11	0.12997 (17)	0.6026 (5)	0.16224 (16)	0.0775 (9)	
H11A	0.1668	0.5252	0.1844	0.093*	
H11B	0.1518	0.7254	0.1576	0.093*	
O4	0.07285 (19)	0.6183 (4)	0.20375 (14)	0.1263 (13)	
H4O	0.049 (3)	0.532 (10)	0.207 (3)	0.190*	

### Atomic displacement parameters (Å^2^)

**Table:**

	*U*^11^	*U*^22^	*U*^33^	*U*^12^	*U*^13^	*U*^23^
O1	0.0538 (10)	0.1121 (17)	0.0539 (10)	0.0251 (9)	−0.0090 (8)	−0.0026 (10)
O2	0.0809 (11)	0.0353 (8)	0.0632 (10)	0.0006 (7)	0.0116 (8)	0.0062 (7)
S1	0.0454 (3)	0.0439 (3)	0.0411 (3)	0.00854 (19)	0.0022 (2)	−0.0011 (2)
C1	0.0384 (9)	0.0358 (9)	0.0404 (10)	0.0034 (7)	0.0051 (8)	−0.0069 (8)
C2	0.0441 (11)	0.0514 (12)	0.0548 (13)	−0.0088 (9)	0.0050 (9)	−0.0136 (10)
C3	0.0587 (13)	0.0475 (12)	0.0664 (15)	−0.0128 (10)	0.0219 (11)	−0.0084 (11)
C4	0.0680 (14)	0.0399 (11)	0.0508 (12)	0.0061 (9)	0.0193 (11)	−0.0007 (9)
C7	0.116 (3)	0.080 (2)	0.0577 (16)	0.0089 (18)	0.0318 (16)	0.0138 (15)
C5	0.0536 (12)	0.0520 (12)	0.0447 (11)	0.0054 (10)	−0.0009 (9)	−0.0071 (10)
C6	0.0383 (10)	0.0427 (11)	0.0449 (11)	−0.0004 (8)	0.0026 (8)	−0.0057 (8)
N1	0.0449 (9)	0.0390 (9)	0.0447 (9)	−0.0018 (6)	0.0129 (7)	−0.0056 (7)
C8	0.0488 (11)	0.0580 (13)	0.0418 (11)	0.0102 (9)	0.0038 (9)	−0.0003 (10)
C9	0.0537 (13)	0.0734 (16)	0.0634 (15)	−0.0154 (12)	0.0206 (11)	−0.0090 (13)
O3	0.0879 (13)	0.0678 (12)	0.0484 (10)	−0.0134 (9)	0.0284 (9)	0.0010 (8)
C10	0.0765 (15)	0.0487 (13)	0.0548 (14)	−0.0185 (11)	0.0248 (12)	−0.0147 (11)
C11	0.0856 (19)	0.080 (2)	0.0669 (18)	−0.0254 (15)	0.0062 (14)	−0.0239 (15)
O4	0.171 (3)	0.116 (2)	0.0927 (17)	−0.0743 (19)	0.0787 (18)	−0.0682 (16)

### Geometric parameters (Å, º)

**Table:**

O1—S1	1.4220 (18)	C6—H6	0.9300
O2—S1	1.4291 (18)	N1—C8	1.465 (3)
S1—N1	1.6252 (17)	N1—C10	1.477 (3)
S1—C1	1.761 (2)	C8—C9	1.503 (3)
C1—C2	1.388 (3)	C8—H8A	0.9700
C1—C6	1.385 (3)	C8—H8B	0.9700
C2—C3	1.375 (4)	C9—O3	1.413 (3)
C2—H2	0.9300	C9—H9A	0.9700
C3—C4	1.392 (4)	C9—H9B	0.9700
C3—H3	0.9300	O3—H3O	0.75 (4)
C4—C5	1.385 (3)	C10—C11	1.454 (4)
C4—C7	1.504 (3)	C10—H10A	0.9700
C7—H7A	0.9600	C10—H10B	0.9700
C7—H7B	0.9600	C11—O4	1.319 (4)
C7—H7C	0.9600	C11—H11A	0.9700
C5—C6	1.382 (3)	C11—H11B	0.9700
C5—H5	0.9300	O4—H4O	0.76 (7)
			
O1—S1—O2	120.20 (12)	C8—N1—S1	117.52 (14)
O1—S1—N1	107.31 (11)	C10—N1—S1	116.78 (14)
O2—S1—N1	106.67 (10)	N1—C8—C9	112.75 (19)
O1—S1—C1	107.29 (10)	N1—C8—H8A	109.0
O2—S1—C1	107.90 (10)	C9—C8—H8A	109.0
N1—S1—C1	106.77 (9)	N1—C8—H8B	109.0
C2—C1—C6	120.13 (19)	C9—C8—H8B	109.0
C2—C1—S1	120.15 (17)	H8A—C8—H8B	107.8
C6—C1—S1	119.70 (15)	O3—C9—C8	109.9 (2)
C3—C2—C1	119.6 (2)	O3—C9—H9A	109.7
C3—C2—H2	120.2	C8—C9—H9A	109.7
C1—C2—H2	120.2	O3—C9—H9B	109.7
C2—C3—C4	121.3 (2)	C8—C9—H9B	109.7
C2—C3—H3	119.3	H9A—C9—H9B	108.2
C4—C3—H3	119.3	C9—O3—H3O	107 (3)
C5—C4—C3	118.0 (2)	C11—C10—N1	114.8 (2)
C5—C4—C7	120.6 (3)	C11—C10—H10A	108.6
C3—C4—C7	121.3 (2)	N1—C10—H10A	108.6
C4—C7—H7A	109.5	C11—C10—H10B	108.6
C4—C7—H7B	109.5	N1—C10—H10B	108.6
H7A—C7—H7B	109.5	H10A—C10—H10B	107.6
C4—C7—H7C	109.5	O4—C11—C10	120.6 (3)
H7A—C7—H7C	109.5	O4—C11—H11A	107.2
H7B—C7—H7C	109.5	C10—C11—H11A	107.2
C6—C5—C4	121.5 (2)	O4—C11—H11B	107.2
C6—C5—H5	119.2	C10—C11—H11B	107.2
C4—C5—H5	119.2	H11A—C11—H11B	106.8
C5—C6—C1	119.36 (19)	C10—C11—H4O	105 (2)
C5—C6—H6	120.3	H11A—C11—H4O	99.7
C1—C6—H6	120.3	H11B—C11—H4O	129.5
C8—N1—C10	117.96 (18)	C11—O4—H4O	115 (5)
			
O1—S1—C1—C2	−22.1 (2)	C2—C1—C6—C5	−0.7 (3)
O2—S1—C1—C2	−152.92 (17)	S1—C1—C6—C5	177.53 (16)
N1—S1—C1—C2	92.73 (18)	O1—S1—N1—C8	−159.22 (16)
O1—S1—C1—C6	159.73 (17)	O2—S1—N1—C8	−29.17 (18)
O2—S1—C1—C6	28.87 (19)	C1—S1—N1—C8	86.00 (16)
N1—S1—C1—C6	−85.47 (17)	O1—S1—N1—C10	52.0 (2)
C6—C1—C2—C3	1.2 (3)	O2—S1—N1—C10	−177.94 (17)
S1—C1—C2—C3	−177.04 (17)	C1—S1—N1—C10	−62.76 (19)
C1—C2—C3—C4	−0.4 (3)	C10—N1—C8—C9	−119.1 (2)
C2—C3—C4—C5	−0.9 (3)	S1—N1—C8—C9	92.5 (2)
C2—C3—C4—C7	179.5 (2)	N1—C8—C9—O3	63.5 (3)
C3—C4—C5—C6	1.4 (3)	C8—N1—C10—C11	94.0 (3)
C7—C4—C5—C6	−179.0 (2)	S1—N1—C10—C11	−117.4 (2)
C4—C5—C6—C1	−0.6 (3)	N1—C10—C11—O4	−54.6 (4)

### Hydrogen-bond geometry (Å, º)

Cg1 is the centroid of the C1–C6 benzene ring.

**Table:**

*D*—H···*A*	*D*—H	H···*A*	*D*···*A*	*D*—H···*A*
O4—H4*O*···O3	0.76 (7)	1.89 (7)	2.634 (3)	166 (7)
O3—H3*O*···O4^i^	0.75 (4)	1.92 (4)	2.661 (3)	172 (4)
C10—H10*A*···O1^ii^	0.97	2.61	3.361 (3)	135
C3—H3···*Cg*1^iii^	0.93	2.78	3.522 (2)	138

Symmetry codes: (i) −*x*, *y*−1/2, −*z*+1/2; (ii) −*x*+1/2, *y*+1/2, *z*; (iii) *x*, −*y*−1/2, *z*−1/2.
